# Mechanical Perturbations of the Walking Surface Reveal Unaltered Axial Trunk Stiffness in Chronic Low Back Pain Patients

**DOI:** 10.1371/journal.pone.0157253

**Published:** 2016-06-16

**Authors:** Maarten R. Prins, Peter van der Wurff, Onno G. Meijer, Sjoerd M. Bruijn, Jaap H. van Dieën

**Affiliations:** 1 Research and Development, Military Rehabilitation Centre ‘Aardenburg’, Doorn, The Netherlands; 2 MOVE Research Institute Amsterdam, Faculty of Behavioural and Human Movement Sciences, Vrije Universiteit Amsterdam, Amsterdam, The Netherlands; 3 Department of Physical Therapy, HU University of Applied Sciences Utrecht, Utrecht, The Netherlands; 4 Orthopaedic Biomechanics Laboratory, Fujian Medical University, Quanzhou, Fujian, PR China; Semmelweis University, HUNGARY

## Abstract

**Introduction:**

Patients with chronic low back pain (CLBP) often demonstrate altered timing of thorax rotations in the transverse plane during gait. Increased axial trunk stiffness has been claimed to cause this movement pattern.

**Objectives:**

The objective of this study was to assess whether axial trunk stiffness is increased in gait in CLBP patients.

**Methods:**

15 CLBP patients and 15 healthy controls walked on a treadmill that imposed rotational perturbations in the transverse plane. The effect of these perturbations on transverse pelvis, thorax and trunk (thorax relative to pelvis) rotations was evaluated in terms of residual rotations, *i*.*e*., the deviation of these movements from the unperturbed patterns. In view of the heterogeneity of the CLBP group, we additionally performed a subgroup comparison between seven patients and seven controls with maximal between-group contrast for timing of thorax rotations.

**Results:**

Rotations of the walking surface had a clear effect on transverse pelvis, thorax and trunk rotations in all groups. No significant between-group differences on residual transverse pelvis, thorax and trunk rotations were observed.

**Conclusion:**

Axial trunk stiffness in gait does not appear to be increased in CLBP. Altered timing of thorax rotations in CLBP does not seem to be a result of increased axial trunk stiffness.

## Introduction

Gait changes in chronic low back pain (CLBP) are generally considered to be responses to the symptoms of low-back pain, irrespective of underlying pathology. Examples of such adaptations are that patients with CLBP prefer a lower gait speed, [1–6] take shorter steps, [1, 2, 4] and demonstrate an altered timing of transverse plane thorax rotations [7].

If a person walks slow, transverse thorax and pelvis rotations are in-phase, *i*.*e*., synchronous rotation in the same direction. When speeding up, the timing of pelvis rotations relative to the pendular movements of the legs changes from out of phase, *i*.*e*., in the opposite direction, to in phase. In healthy subjects, the timing of thorax rotations relative to the legs remains constantly out of phase over gait speeds. The result is a clear shift towards out-of phase pelvis-thorax coordination. On a group level this shift in movement pattern occurs less in CLBP patients [1–5, 8], because they show a gradual change in thorax timing towards in-phase with the legs with increasing gait speed [7]. Not all CLBP patients demonstrate this behaviour. Studies that found significant between group differences in transverse pelvis-thorax coordination reported a high within-group variance [2, 3, 7].

The underlying mechanism of altered timing of thorax rotations in CLBP gait remains unknown. One way to attenuate out-of-phase movement of pelvis and thorax would be to alter the axial stiffness of the trunk [9]. By enhancing axial trunk stiffness, through co-contraction or through increasing reflex gains of trunk muscles, the spine will be protected against large movement excursions due to mechanical perturbations. Several studies that investigated trunk kinematics in CLBP gait reported signs of increased axial trunk stiffness in these patients in terms of altered timing of transverse thorax rotations and decreased variability of transverse trunk rotations [2, 3, 6]. However, experimentally stiffening the spine of healthy subjects did not result in the same alteration of thorax timing at high gait speeds as seen in patients [10].

The objective of this study was to assess whether axial trunk stiffness is increased in gait in patients with CLBP. To this end, the effects of external perturbations on transverse pelvis and thorax rotations in CLBP patients and healthy controls were assessed. In view of the heterogeneity of the patient population, we also performed a subgroup analysis, comparing CLBP patients with the thorax moving most in-phase with the movements of the legs on one hand to the healthy controls with the thorax moving most out-of-phase with the legs on the other hand. We expected that CLBP patients would demonstrate increased axial trunk stiffness. We therefore hypothesised that rotational perturbations of the walking surface would perturb the pelvis less in CLBP patients compared to healthy controls (since in CLBP patients effective inertia of the pelvis would be higher due to stronger coupling between pelvis and thorax), but the thorax more. We expected all between-group differences to be more pronounced when comparing CLBP patients with more in-phase rotations of thorax and legs to healthy controls with more out-of-phase movements of those segments.

## Materials and Methods

All measurements were performed at the Military Rehabilitation Centre Aardenburg, Doorn, The Netherlands. The protocol of this study was approved by the Medical Ethical Committee of the VU Medical Centre, Amsterdam, The Netherlands (NL 37399.029.12).

### Participants

Fifteen male CLBP patients were recruited from the in- and outpatient population of the rehabilitation centre. Fifteen healthy male controls were recruited from the personnel of the centre and by word of mouth in the local community. All participants had to be between 20 and 50 years of age, and have no condition that might interfere with gait (other than CLBP in the CLBP patients), or any condition that rendered them unfit to be tested. CLBP patients had to have experienced low back pain in the three months prior to inclusion and have a minimum current Visual Analogue Score (VAS) for low back pain intensity of 20 mm at the time of inclusion. Patients were excluded if they had undergone spinal surgery. Healthy controls were only included if they had no self-reported history of back pain episodes lasting more than six weeks and had not experienced back pain during the last three months. Inclusion occurred at least two weeks before the experimental procedures took place. All participants were asked not to take any pain relieving medication 24 hours prior to testing, and all participants signed an informed consent.

### Experimental procedure

Participants were tested in a Computer Assisted Rehabilitation Environment (CAREN, Motek Forcelink, Amsterdam, The Netherlands). The CAREN system consists of an instrumented treadmill mounted on a six degrees-of-freedom motion platform. Participants completed an unperturbed gait trial and a perturbation trial of two and five minutes respectively. During all trials participants walked at 3.8 km/h (1.06 m/s). To limit head movements, participants were instructed to gaze at non-frightening wildlife pictures projected at eye level.

**Fig 1:**
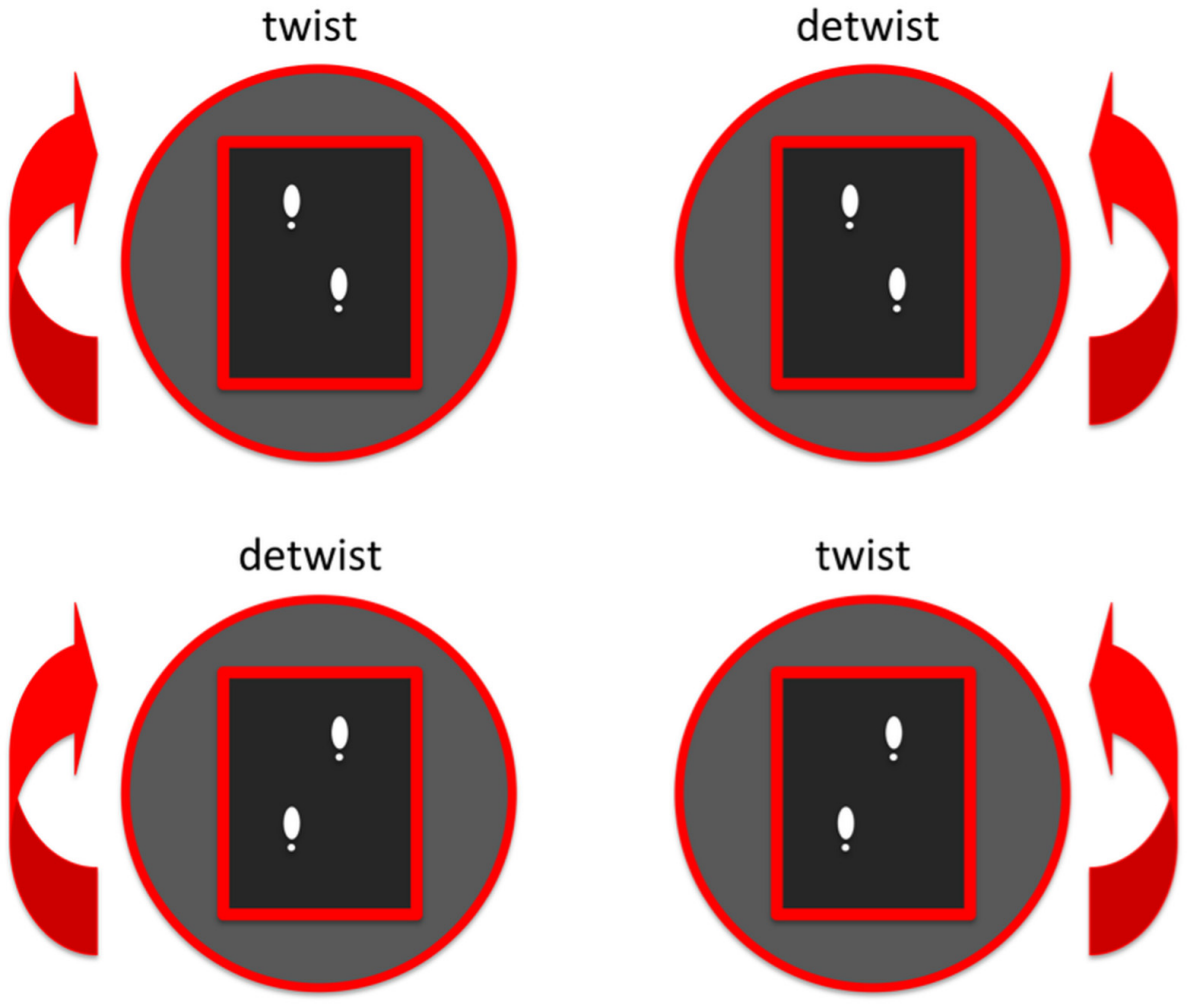
Imposed perturbations. Four perturbations were imposed. Two twisting perturbations that increased trunk excursion and two detwisting perturbations that decreased trunk excursion.

During the perturbation trial, participants were exposed to perturbations of the walking surface. The platform rotated eight degrees to the left or to the right every five to ten seconds. Two seconds after the perturbation, the platform gently rotated back to the neutral orientation. The perturbation was timed in such a way that the rotational velocity of the platform was maximal (28 degrees/s) around heel strike. An accurate timing of perturbations was achieved using a custom-made algorithm in D-flow (Motek Forcelink, Amsterdam, The Netherlands) that predicted the timing of an incoming heel strike based on three preceding heel strikes of the same leg using centre of pressure data. Perturbations to the left and right were imposed with the left leg either leading or trailing, resulting in 4 possible perturbations. These perturbations either increased or decreased trunk rotational excursion. Therefore we will further refer to these perturbations as ‘twisting’, *i*.*e*., increasing trunk excursion and ‘detwisting’, *i*.*e*., decreasing trunk excursion (Fig 1). The exact instant and direction of each individual perturbation was not predictable for the participants. After 48 perturbations (2 leading legs × 2 directions × 12 perturbations), the trial was stopped.

### Data collection

Single markers were placed on both lateral femoral condyles and on both heels. To determine the orientation of pelvis and thorax, a cluster of three reflective markers was attached to the dorsal side of the pelvis at the level of the posterior superior iliac spines and to the dorsal side of the thorax at the level of the 6^th^ thoracic spinous process using neoprene straps. Platform orientation was measured synchronously with the movement of the participant by placing one marker on each corner of the platform. Marker positions were recorded using twelve high-resolution infrared cameras (VICON, Oxford, UK) at a rate of 100 samples/s.

### Data analysis

Marker data were filtered with a fourth order bi-directional (two times second order) low pass Butterworth filter with a cut-off frequency of 10 Hz. Rotations of the platform, pelvis, and thorax around the global vertical axis were expressed relative to their orientation during a calibration trial with the participant standing in the anatomical position. Trunk rotation was calculated as pelvis rotation minus thorax rotation. Heel strikes were determined as local maxima in antero-posterior position of the heel markers.

From the steady-state trial we calculated (inter)segmental ranges of motion (ROM) and relative timing of segmental movements. Pelvis, thorax and trunk ROMs were calculated by subtracting the maximal transverse rotation from the minimal value per stride and then averaging over strides. Relative timing of transverse pelvis and thorax rotations and antero-posterior excursion of the left knee, *i*.*e*., leg-pelvis, leg-thorax and pelvis-thorax was expressed as average continuous relative Fourier phase (RFP) by subtracting the Fourier phase of the lower segment from that of the higher segment. An RFP of zero degrees corresponds to perfect synchronous movement of both segments and an RFP of 180 degrees to perfect asynchronous movement of both segments [11].

The effect of platform perturbations on transverse (inter)segmental rotations was assessed by calculating ‘residual rotations’. The residual rotations of the pelvis, thorax and trunk were obtained by time-normalizing transverse segmental rotations to stride-cycle duration, then subtracting the average value calculated over the unperturbed trial from each individual stride and then de-normalizing these residual rotations back to trial-time. Residual segmental rotations were assessed over a time window from 1 second before maximal platform velocity to 1 second after maximal platform velocity.

### Statistical analysis

All statistical analyses were performed twice; once comparing the entire CLBP group to the entire control group and once comparing two subgroups: *CLBP+*, the seven patients with the lowest leg-thorax RFP and *control+*, the seven controls with the highest leg-thorax RFP. Participants’ age, height and weight were compared between groups using independent sample t-tests. RFPs are circular outcomes, and these values were compared between groups using a two-sample Watson-Williams test [12]. To evaluate the effect of perturbations on segmental residual rotations we used one dimensional statistical parametric mapping (SPM1D) [13]. To check if the perturbations had an effect on segmental rotations and to make sure that segmental rotations were not significantly different from unperturbed gait, i.e., residual rotation equal to zero, right before the perturbation was induced, six SPM1D one sample *t*-tests were used, i.e., 3 segments x 2 perturbation types. To test for differences between groups a SPM1D mixed model ANOVA with perturbation type, i.e., twist/detwist, as within-subjects factor, and group, i.e., CLBP/Control as between-subject factor and residual segmental rotation as independent variable was performed for each segment. Circular means, circular standard deviations and the Watson-Williams tests were calculated using the CircStat toolbox in MATLAB [12]. The independent sample t-tests were performed using Statistical Package for the Social Sciences (SPSS), version 22 and the one dimensional *t* statistic was calculated using the spm1d package version 0.3.1 in MATLAB. The alpha level of each test was set to 0.05.

## Results

At the time of the experiment, CLBP patients reported a mean VAS for current pain of 21 (SD 17) mm and the CLBP+ group of 17 (SD 8) mm. None of the patients showed signs of radicular pain. The pain intensity of the CLBP patients in the CLBP+ group was not significantly different from the other CLBP patients. At the time of the experiment, i.e., two weeks after inclusion, 8 CLBP patients had a VAS for current pain below 20 mm (5 in the CLBP+ group), but none were pain-free. There were no significant differences in age, height or weight between groups. All participants completed the entire experimental procedure (Table 1).

**Table 1:** Subject characteristics.

	Control	CLBP	p	Control+	CLBP+	p
**n (m/f)**	15 (5/0)	15 (15/0)	-	7 (7/0)	7 (7/0)	-
**Age in years**	33 (6)	34 (11)	NS	30 (4)	38 (11)	NS
**Height in cm**	186 (11)	184 (8)	NS	185 (12)	186 (9)	NS
**Weight in kg**	80 (14)	84 (9)	NS	79 (16)	89 (11)	NS
**VAS Pain in mm**	0 (0)	21.4 (17)	-	0 (0)	17 (8)	-

NS: Not Significant (p-value > .05)

### Segmental rotation characteristics during unperturbed gait

**Fig 2:**
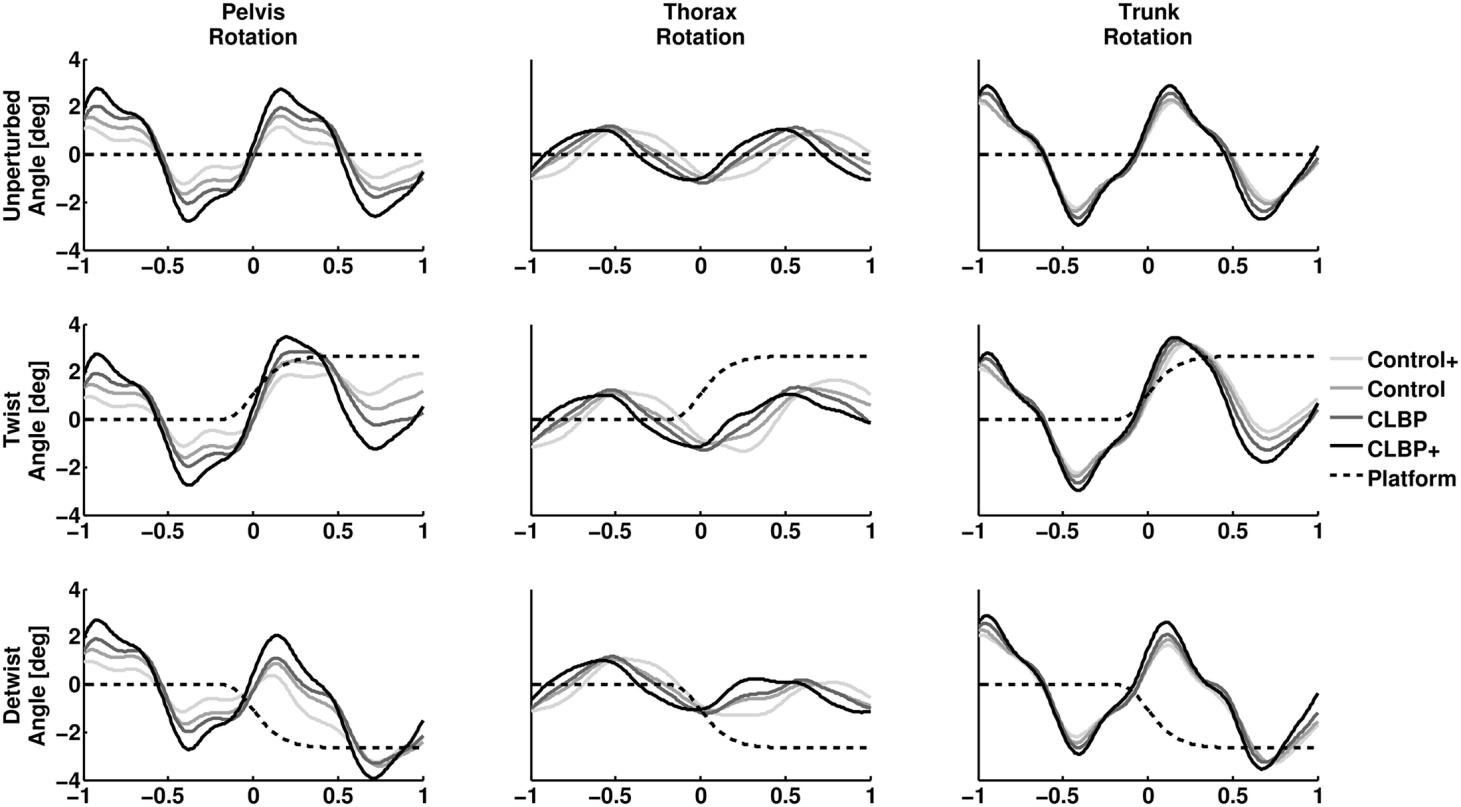
Overview of the average effect of platform perturbations on segmental rotations. Average pelvis (left panel), thorax (middle panel) and trunk (right panel) rotations during unperturbed gait (upper panel), twisting perturbations (middle panel) and detwisting perturbations (lower panel) are shown. The horizontal axis displays time to maximal platform velocity in seconds. The vertical axis displays segmental rotation in degrees.

The average unperturbed segmental rotations in the upper panel of Fig 2 show clear periodic movements for all segments in all groups. Maximal thorax rotation occurred around heel strike in all groups with the thorax maximally rotated to the right around left heel strike and to the left around right heel strike. Trunk rotations peaked 100-200ms after heel strike, which coincided with maximal pelvis excursion.

The average pelvis-thorax RFP was lower in the CLBP group than the control group, although not significantly. Ranges of motion were not significantly different between the CLBP and control group. The pelvis-thorax and leg-thorax RFP were significantly lower in the CLBP+ group than in the control+ group, but no significant difference in leg-pelvis RFP between these groups was observed. Pelvis ROM was significantly higher in the CLBP+ group compared to the control+ group (Table 2).

**Table 2:** Mean (SD) transverse segmental rotation characteristics during unperturbed gait.

	Control	CLBP	p	Control+	CLBP+	p
**Pelvis ROM in deg**	4.9 (1.6)	5.3 (2.0)	NS	3.8 (.7)	6.4 (2.1)	.009
**Thorax ROM in deg**	3.4 (1.0)	3.2 (1.5)	NS	3.2 (1.2)	3.0 (2.0)	NS
**Trunk ROM in deg**	6.0 (2.2)	6.3 (2.2)	NS	5.4 (1.4)	7.0 (2.9)	NS
**Pelvis-Thorax RFP in deg**	101 (39)	83 (26)	NS	131 (37)	69 (19)	.004
**Leg-Thorax RFP in in deg**	240 (37)	219 (28)	NS	271 (16)	195 (13)	< .001
**Leg-Pelvis RFP in deg**	133 (33)	136 (20)	NS	137 (41)	126 (16)	NS

### Perturbation outcomes

**Fig 3:**
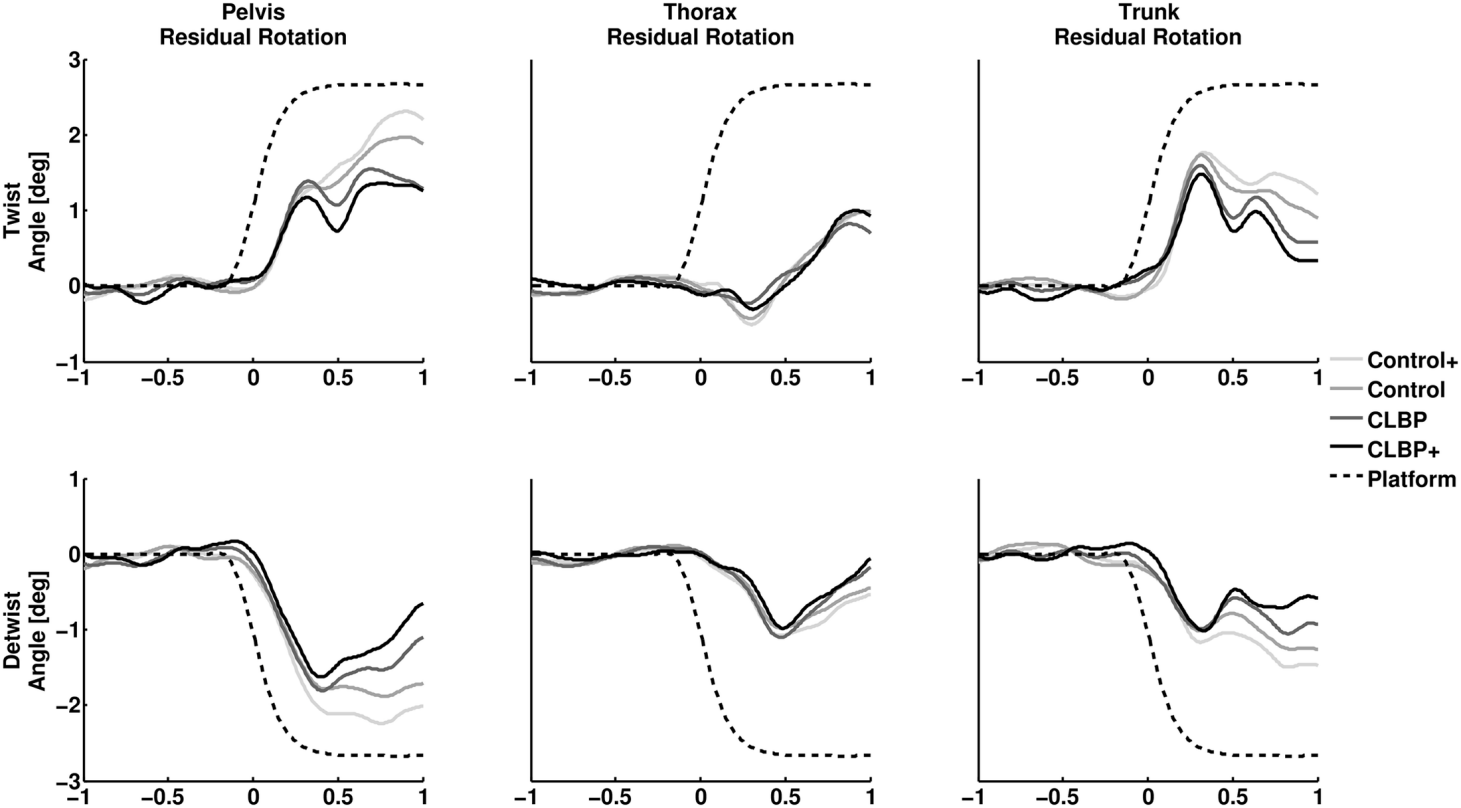
Overview of the average effect of platform perturbations on residual segmental rotations. Residual pelvis (left panel), thorax (middle panel) and trunk (right panel) rotations during, twisting perturbations (upper panel) and detwisting perturbations (lower panel) are shown. The horizontal axis displays time to maximal platform velocity in seconds. The vertical axis displays residual segmental rotation in degrees. Note that values for platform excursion are scaled down by a factor three.

The effect of perturbations on transverse segmental rotations is displayed in Figs 2 and 3. The onset of platform perturbations occurred approximately 150 milliseconds before maximal platform velocity. None of the residual segmental rotations were significant at this instant. Both twisting and detwisting perturbations resulted in significant residual rotations of pelvis, thorax and trunk (all p < .01), see S1 and S2 Figs. None of the SPM1D mixed model ANOVAs comparing residual pelvis, thorax and trunk rotations between groups, around platform perturbations, yielded a significant group effect. In other words, the movements displayed in Fig 3 are not significantly different between CLBP and control nor CLBP+ and control+ at any instant displayed. The results of the SPM1D mixed model ANOVAs are presented in S3 and S4 Figs.

## Discussion

We studied whether axial trunk stiffness is increased in gait in patients with CLBP by assessing the effects of transverse mechanical perturbations of the walking surface on pelvis and thorax rotations during gait. Both twisting and detwisting perturbations had a clear effect on transverse pelvis rotations in all participants, so the perturbation of the pelvis via the walking surface was successful. The effect of perturbations on residual pelvis, thorax and trunk rotations was similar across groups. Therefore, axial trunk stiffness does not appear to be altered in gait in CLBP. Hence this would not explain changes timing of transverse thorax rotations during gait in CLBP. Because altered timing of thorax rotations is not consistently observed in CLBP patients, and in fact was not significant in our patient group, we performed a sub-group analysis, comparing healthy controls with a relatively high leg-thorax RFP to CLBP patients with a relatively low leg-thorax RFP. The timing of thorax rotations during unperturbed gait was significantly different between these groups whereas timing of pelvis rotations was not. We did not find significant differences in perturbation effects between these groups either. Therefore, altered timing of transverse thorax rotations in gait in CLBP does not appear to be the result of increased intrinsic axial trunk stiffness.

Because changes in timing of transverse thorax rotations during gait in CLBP do not seem to be caused by altered axial trunk stiffness, the question arises how CLBP patients do alter the timing of thorax rotations. One explanation is that differences in reaction forces in the shoulders, as a result of arm swing, alter timing of thorax rotations. Although Huang et al. [7] found no significant group differences in timing or amplitude of the antero-posterior movements of the wrist in CLBP compared to healthy controls, they did not calculate net reaction forces in the shoulders. Because the arms contribute considerably to the bodies’ angular momentum [14], small deviations in movements of the arms may have large effect on movements of the thorax. Another possible explanation is that the amplitude of transverse pelvis rotations might influence timing of transverse thorax rotations. We observed that pelvis excursions in the CLBP+ group were nearly twice as large as those in the control+ group, even though we selected these two groups based on timing of the thorax. Increased amplitudes of pelvis rotations during unperturbed gait have been reported in patients with lumbar disc herniation [7] and pelvic girdle pain [15]. Why these patients walk with larger transverse pelvis rotations and how this might influence timing of thorax rotations remains unknown.

Besides altered kinematics in CLBP gait, other aspects of CLBP have been related to increased trunk stiffness in the literature. A recent systematic review reported that the average activity of several trunk muscles is elevated in gait in CLBP patients [16]. Co-contraction of trunk muscles can result in increased trunk stiffness [17]. However, increased activity of a trunk muscle will not increase stiffness in all movement directions of the trunk (e.g. flexion/extension and rotations) equally [18]. An increase in axial trunk stiffness will mainly be caused by the external and internal obliques [18]. In the review of Ghamkhar and Kahlaee [16] increased activity was reported mainly for trunk muscles acting in the sagittal plane. Only one included study assessed the activity of a clear trunk rotator (external oblique) and found no increased average activation over strides relative to a control group [19]. Based on these results one would expect increased trunk stiffness in the sagittal, but not the transverse plane.

Visual inspection of residual rotations in response to platform perturbations (Fig 3) reveals that, on average, some, albeit non-significant, between group differences in residual pelvis and trunk rotations started to occur 300 ms after maximal platform velocity. Such differences would be too late to be attributed to differences in stiffness, and might be the result of subtle group differences in late, possibly voluntary responses to the perturbation [20].

This study has several limitations that need to be addressed. First, we did not estimate actual axial trunk stiffness in our subjects. It is conceivable that kinematics in reaction to perturbations between groups can be similar despite differences in axial trunk stiffness. This would imply a higher effective inertia of the thorax resulting from differences in mass or amplitude of arm-swing. The mass of our subjects did not differ significantly between groups and we found no indications of differences in arm-swing (results not presented). Therefor, the measurements of kinematics alone seem sufficient to determine if considerable differences in axial trunk stiffness exist between groups. Second, we used only male participants. Women are known to walk with larger transverse pelvis and thorax rotations [21]. Although we found the same pelvis-thorax coordination patterns as previous studies that did include women in their sample [2], we cannot be sure that this coordination pattern is achieved in the same way in both sexes. Another issue is the type of perturbation used to impose a rotation of the pelvis. Although a perturbation of the walking surface enables participants to walk with fewer constraints compared to experiments with perturbations imposed directly to the thorax or pelvis, the effect of our perturbation on pelvis rotations depends on the mechanics of the legs. Fourth, there is a possibility that small consistent differences in axial trunk stiffness exist between groups that were not detected due to a relatively small sample size. However, all between group differences were small as can be seen in Figs 2 and 3 and group effects remained far from statistical significance (S1 and S2 Figs). Finally, the duration of the perturbations was quite long (ca. 0.5 seconds), due to properties of the system used. Therefore, it is possible that measured residual rotations are partially influenced by voluntary muscle activity, which can occur within 200 milliseconds [22]. Residual rotations of the thorax are expected to be in the same direction as the platform. However, directly after the onset of twisting perturbations, residual thorax rotations were in opposite direction in all groups (although not significant), which may suggest an active response.

## Conclusions

Our results suggest that axial trunk stiffness is not increased in gait in patients with CLBP. Altered timing of thorax rotations does not seem to be a result of altered trunk stiffness.

## Supporting Information

S1 Data Individual data points. The individual data points behind presented summary statistics.
(CSV)

S1 Fig One dimensional one sample t-test of residual segmental rotations of the control group compared to the CLBP group. The one dimensional t-statistic of residual pelvis (left panel), thorax (middle panel) and trunk (right panel) rotations of the control group compared to the CLBP group during twisting (upper panel) and detwisting perturbations (lower panel). The horizontal axis displays time to maximal platform velocity in seconds. The vertical axis displays the one dimensional t-statistic. At instances where the black line is above the dotted red line, the residual segmental rotations are significantly different from zero.
(TIF)

S2 Fig One dimensional one sample t-test of residual segmental rotations of the control+ group compared to the CLBP+ group. The one dimensional t-statistic of residual pelvis (left panel), thorax (middle panel) and trunk (right panel) rotations of the control+ group compared to the CLBP+ group during twisting (upper panel) and detwisting perturbations (lower panel). The horizontal axis displays time to maximal platform velocity in seconds. The vertical axis displays the one dimensional t-statistic. At instances where the black line is above the dotted red line, the residual segmental rotations are significantly different from zero.
(TIF)

S3 Fig One dimensional repeated measures ANOVA of residual segmental rotations of the control group compared to the CLBP group. The one dimensional F-statistic of residual pelvis (upper panel), thorax (middle panel) and trunk (lower panel) rotations of the control group compared to the CLBP group during perturbations. The effect of group (left panel), perturbation type (middle panel) and group x perturbation type interaction (right panel) are displayed. The horizontal axis displays time to maximal platform velocity in seconds. The vertical axis displays the one dimensional F-statistic. A significant effect is present at instances where the black line is above the dotted red line.
(TIF)

S4 Fig One dimensional repeated measures ANOVA of residual segmental rotations of the control+ group compared to the CLBP+ group. The one dimensional F-statistic of residual pelvis (upper panel), thorax (middle panel) and trunk (lower panel) rotations of the control+ group compared to the CLBP+ group during perturbations. The effect of group (left panel), perturbation type (middle panel) and group x perturbation type interaction (right panel) are displayed. The horizontal axis displays time to maximal platform velocity in seconds. The vertical axis displays the one dimensional F-statistic. A significant effect is present at instances where the black line is above the dotted red line.
(TIF)
