# Supplementary figures and images for: Mechanical Perturbations of the Walking Surface Reveal Unaltered Axial Trunk Stiffness in Chronic Low Back Pain Patients
Source: PLoS One. 2016 Jun 16;11(6):e0157253. doi: 10.1371/journal.pone.0157253 (PMC4911008; doi:10.1371/journal.pone.0157253)

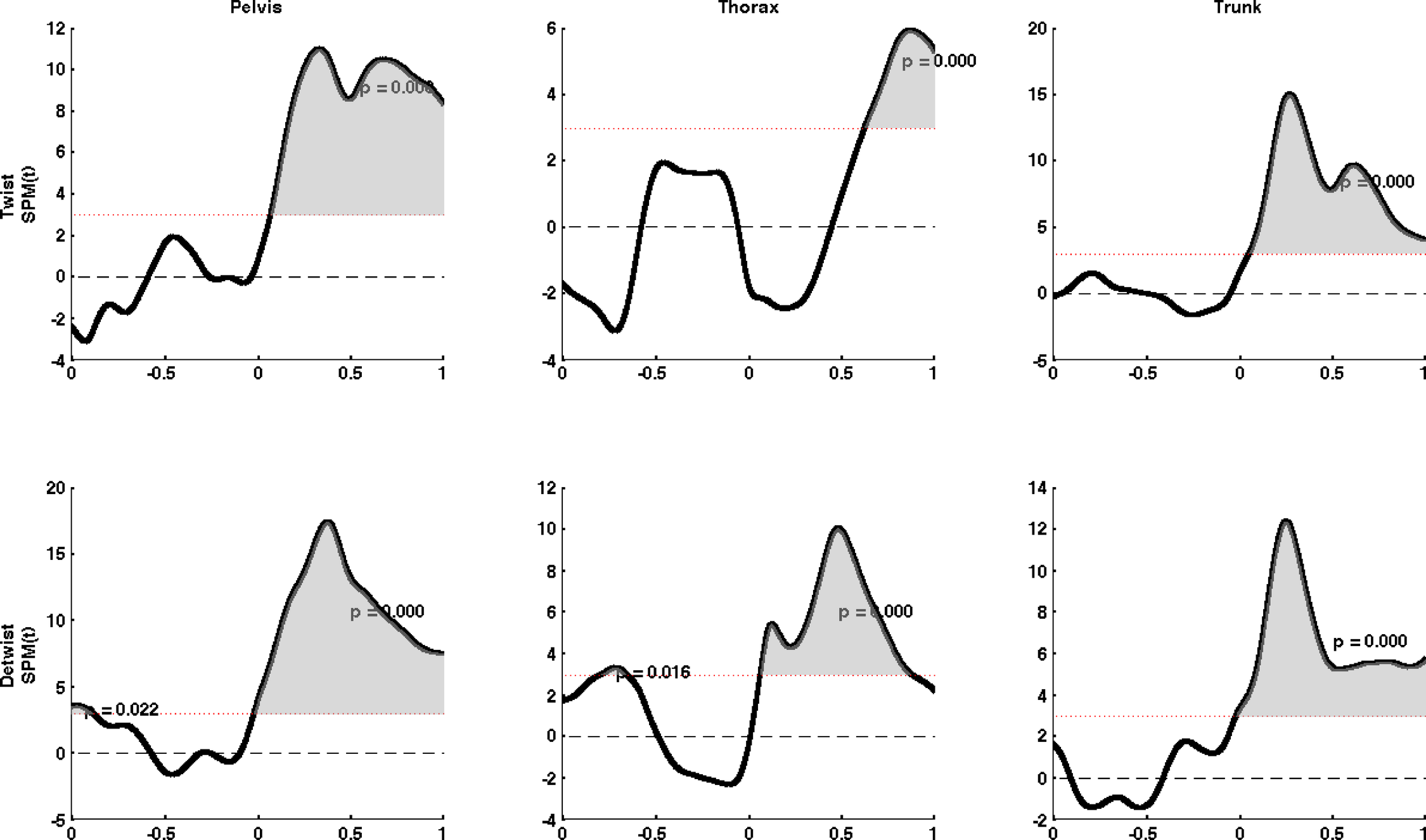

Supplement: S1 Fig — The one dimensional t-statistic of residual pelvis (left panel), thorax (middle panel) and trunk (right panel) rotations of the control group compared to the CLBP group during twisting (upper panel) and detwisting perturbations (lower panel). The horizontal axis displays time to maximal platform velocity in seconds. The vertical axis displays the one dimensional t-statistic. At instances where the black line is above the dotted red line, the residual segmental rotations are significantly different from zero. (TIF) [file pone.0157253.s002.tif]

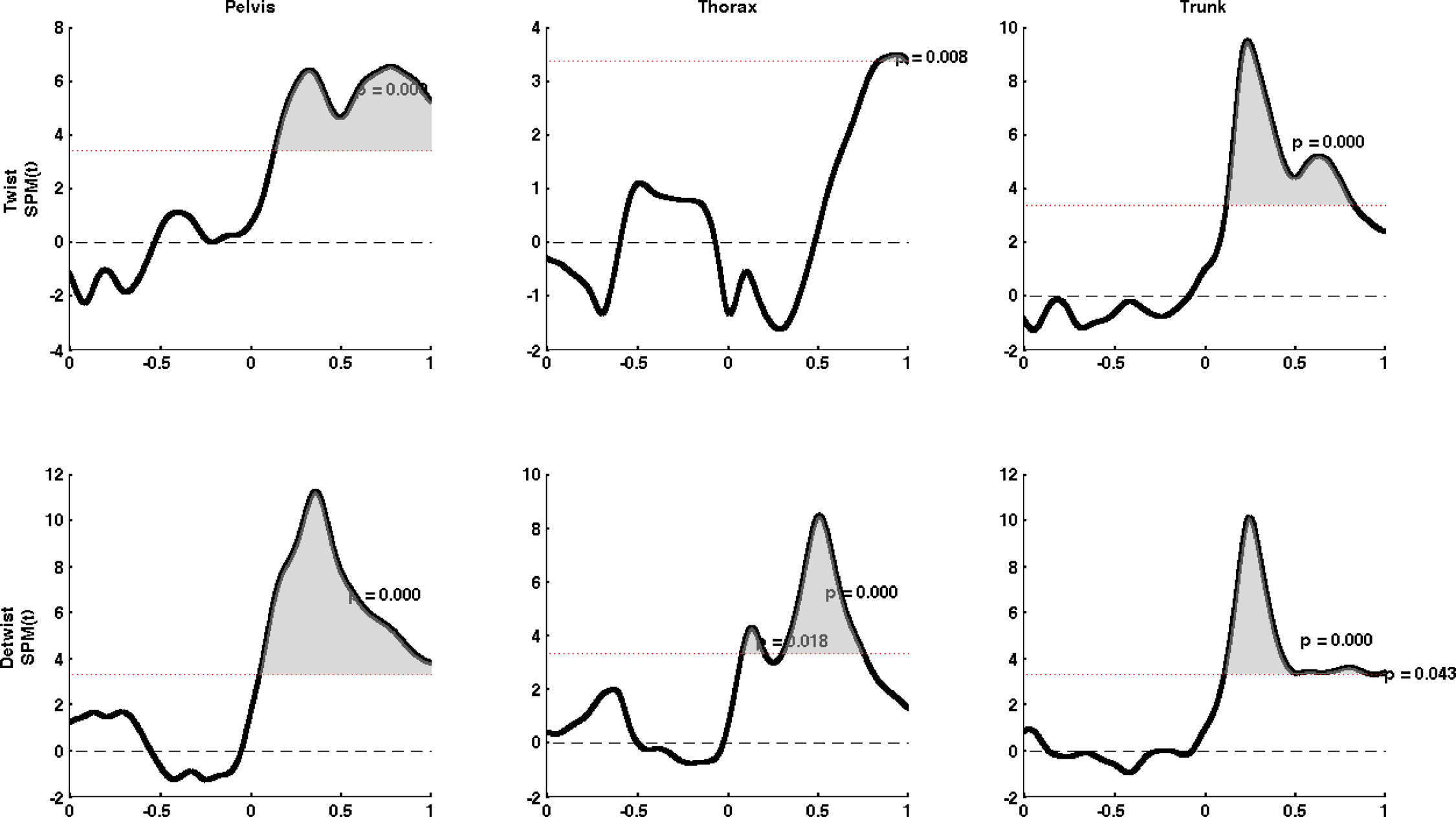

Supplement: S2 Fig — The one dimensional t-statistic of residual pelvis (left panel), thorax (middle panel) and trunk (right panel) rotations of the control+ group compared to the CLBP+ group during twisting (upper panel) and detwisting perturbations (lower panel). The horizontal axis displays time to maximal platform velocity in seconds. The vertical axis displays the one dimensional t-statistic. At instances where the black line is above the dotted red line, the residual segmental rotations are significantly different from zero. (TIF) [file pone.0157253.s003.tif]

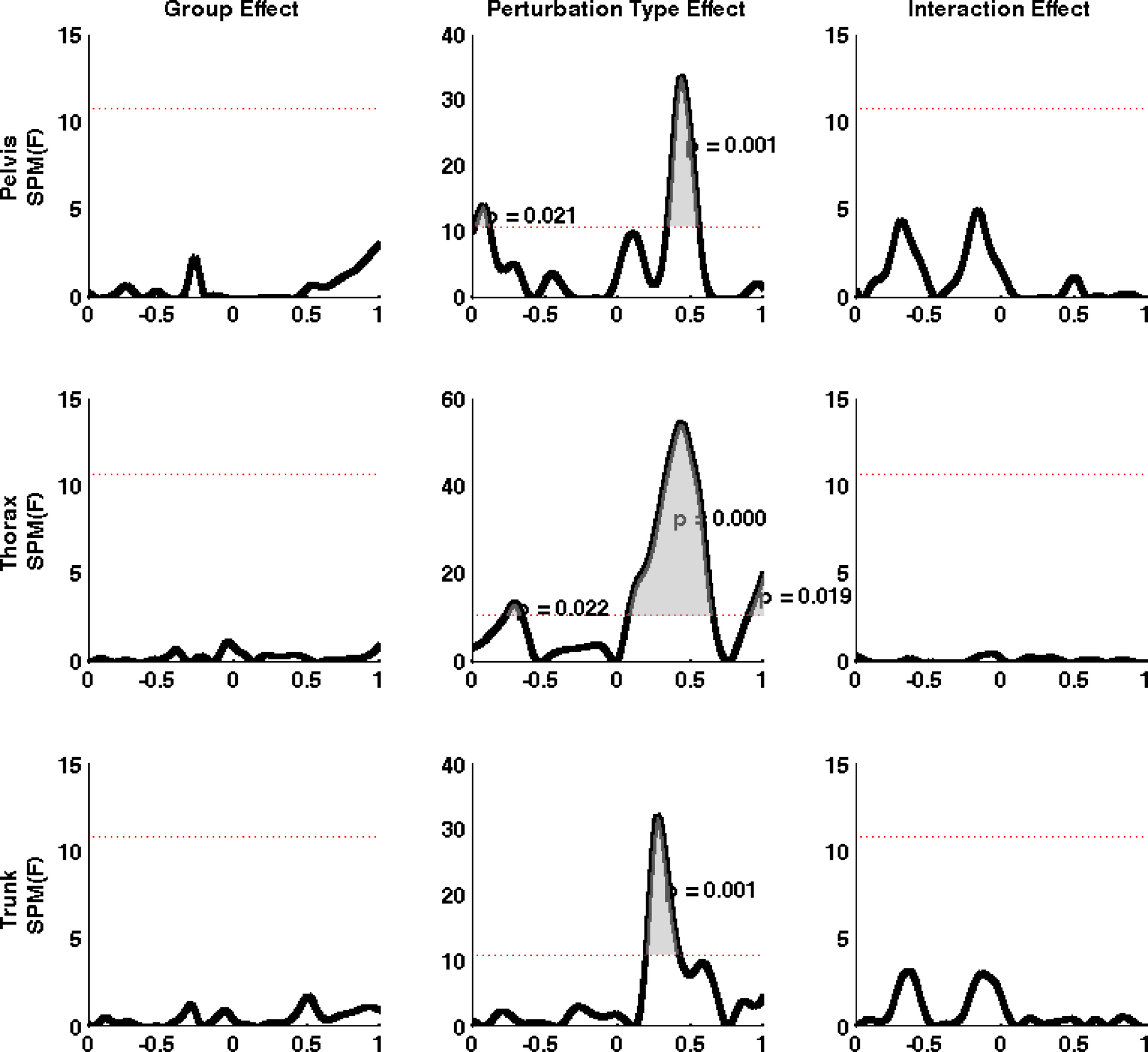

Supplement: S3 Fig — The one dimensional F-statistic of residual pelvis (upper panel), thorax (middle panel) and trunk (lower panel) rotations of the control group compared to the CLBP group during perturbations. The effect of group (left panel), perturbation type (middle panel) and group x perturbation type interaction (right panel) are displayed. The horizontal axis displays time to maximal platform velocity in seconds. The vertical axis displays the one dimensional F-statistic. A significant effect is present at instances where the black line is above the dotted red line. (TIF) [file pone.0157253.s004.tif]

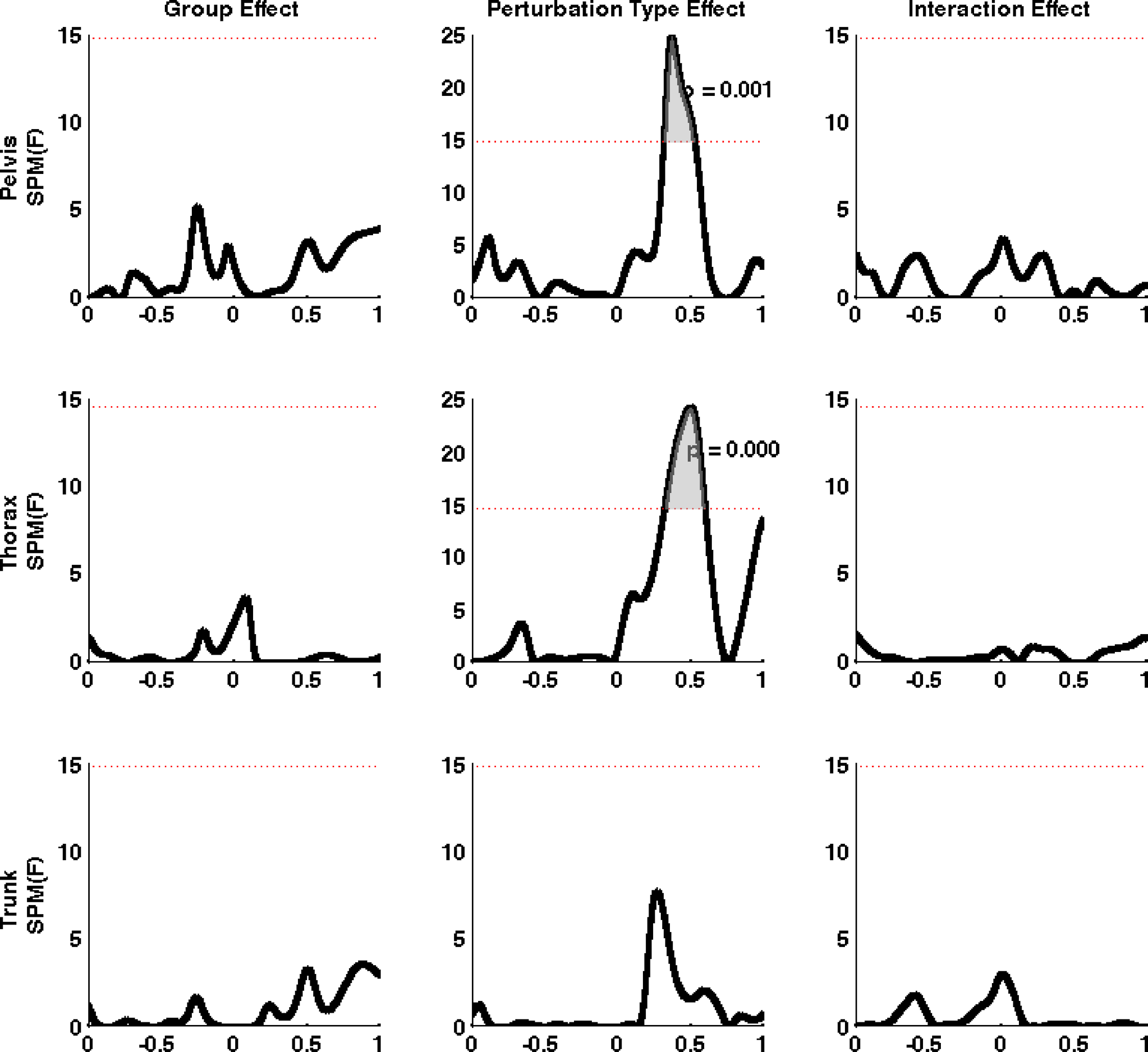

Supplement: S4 Fig — The one dimensional F-statistic of residual pelvis (upper panel), thorax (middle panel) and trunk (lower panel) rotations of the control+ group compared to the CLBP+ group during perturbations. The effect of group (left panel), perturbation type (middle panel) and group x perturbation type interaction (right panel) are displayed. The horizontal axis displays time to maximal platform velocity in seconds. The vertical axis displays the one dimensional F-statistic. A significant effect is present at instances where the black line is above the dotted red line. (TIF) [file pone.0157253.s005.tif]
